# Investigation of rank order centroid method for optimal generation control

**DOI:** 10.1038/s41598-024-61945-z

**Published:** 2024-05-17

**Authors:** T. Varshney, A. V. Waghmare, V. P. Singh, M. Ramu, N. Patnana, V. P. Meena, Ahmad Taher Azar, Ibrahim A. Hameed

**Affiliations:** 1 Department of EECE, Sharda University, Greater Noida, Uttar Pradesh India; 2 Department of Electrical Engineering, Malaviya National Institute of Technology, Jaipur, Rajasthan 302017 India; 3 Department of ECE, GITAM University, Vizag, Andhra Pradesh India; 4 Department of Electrical and Electronics Engineering, Amrita School of Engineering, Amrita Vishwa Vidyapeetham, Bengaluru, India; 5 College of Computer and Information Sciences, Prince Sultan University, Riyadh, 11586 Saudi Arabia; 6 Automated Systems and Soft Computing Lab (ASSCL), Prince Sultan University, Riyadh, Saudi Arabia; 7 Faculty of Computers and Artificial Intelligence, Benha University, Benha, 13518 Egypt; 8 Department of ICT and Natural Sciences, Norwegian University of Science and Technology, Larsgardsvegen, 2, 6009 Alesund, Norway

**Keywords:** MCDM, Rank order centroid, Jaya optimization, PID controller, AGC, Engineering, Electrical and electronic engineering

## Abstract

Multi-criteria decision-making (MCDM) presents a significant challenge in decision-making processes, aiming to ascertain optimal choice by considering multiple criteria. This paper proposes rank order centroid (ROC) method, MCDM technique, to determine weights for sub-objective functions, specifically, addressing issue of automatic generation control (AGC) within two area interconnected power system (TAIPS). The sub-objective functions include integral time absolute errors (ITAE) for frequency deviations and control errors in both areas, along with ITAE of fluctuation in tie-line power. These are integrated into an overall objective function, with ROC method systematically assigning weights to each sub-objective. Subsequently, a PID controller is designed based on this objective function. To further optimize objective function, Jaya optimization algorithm (JOA) is implemented, alongside other optimization algorithms such as teacher–learner based optimization algorithm (TLBOA), Luus–Jaakola algorithm (LJA), Nelder–Mead simplex algorithm (NMSA), elephant herding optimization algorithm (EHOA), and differential evolution algorithm (DEA). Six distinct case analyses are conducted to evaluate controller’s performance under various load conditions, plotting data to illustrate responses to frequency and tie-line exchange fluctuations. Additionally, statistical analysis is performed to provide further insights into efficacy of JOA-based PID controller. Furthermore, to prove the efficacy of JOA-based proposed controller through non-parametric test, Friedman rank test is utilized.

## Introduction

Every decision necessitates a thorough consideration of the decision-making process to enhance its effectiveness. Decision-making serves as a mechanism for problem-solving, integrating various variables to arrive at a favorable outcome^1^. Implicit or explicit assumptions influenced by diverse factors such as physiological, biological, cultural, and social elements can shape this process. Nowadays, the complexity of decision-making problems can be addressed through computer programs, statistical techniques, economic theories, and mathematical equations, offering automated calculation and solution estimation. Among these tools, multi-criteria decision making (MCDM) stands out as widely utilized across various fields^2^.

MCDM involves evaluating, prioritizing, ranking, or selecting alternatives from a finite set of options, taking into account multiple, often conflicting criteria^3^. The weighting of criteria is significant in MCDM models as it reflects the relative importance of the criteria under consideration. The combined impact of all weighted criteria determines the overall performance of the system in question. Additionally, MCDM, also known as multiple criteria decision analysis (MCDA), represents a research field that encompasses the evaluation of diverse criteria within a given context or research domain. MCDA techniques are broadly categorized into two components: non-compensatory and compensatory techniques^4^. Non-compensatory techniques focus solely on better-performing criteria for weight determination, disregarding poorly performing ones. Conversely, compensatory techniques consider both poorly performing and better-performing criteria, with the latter compensating for the former. Rank order centroid (ROC) method is a promising non-compensatory technique for criteria weight determination^5^. ROC method possesses benefits like:

- Simplicity: The ROC method is straightforward and easy to implement, making it accessible to decision-makers with varying levels of expertise.
- Transparency: It provides a clear rationale for criteria weighting, as it considers the relative ranks of alternatives rather than requiring complex calculations or subjective judgments.
- Flexibility: The ROC method can handle both quantitative and qualitative criteria, allowing for a comprehensive assessment of decision alternatives.
- Robustness: It is less sensitive to outliers or extreme values compared to some other MCDM techniques, making it suitable for decision-making in diverse contexts.
- Intuitiveness: The method aligns with human intuition by emphasizing the relative ordering of alternatives based on their performance across criteria, which resonates with decision-makers’ cognitive processes.

Overall, the ROC method offers a practical and effective approach to criteria weighting in MCDM, striking a balance between simplicity, transparency, flexibility, robustness, and intuitiveness^6^.

ROC method, originally proposed by Barren and Barrot, serves as a weight estimation technique that aims to minimize the maximum error associated with each weight assignment^6^. This is achieved by determining the centroid of all potential weight distributions while preserving the rank order of objectives^3^. Various researchers have applied the ROC method across diverse engineering domains for the assessment and prioritization of multiple criteria^7–9^. Esangbedo et al. introduced a relaxed variant of the ROC method in their study^10^, employing it for subcontractor selection in photothermal power station construction projects. In another work by Kim et al.^11^, ROC was utilized alongside other weight determination techniques to assign maintenance demand weights based on traffic load classifications. Ribeiro et al.^12^ utilized the ROC method to select appropriate current transducers for a smart plug project development. Yadav et al. proposed a novel approach involving the use of a grey-wolf optimizer and ROC-based technique to reduce the order of robotic-cycle controller^13^.

Several researchers have integrated MCDA techniques in their studies^14–16^ to rank and determine appropriate weights for each sub-objective function within an overall objective function designed for automatic generation control (AGC) systems. In AGC, key factors essential for maintaining the overall power balance of the system include frequency deviation, area control errors, and tie-line power deviation^17^. Controllers employed in AGC play a crucial role in mitigating imbalances within the system^18–20^. Optimizing controller parameters is vital to ensure reliable and efficient power flow. During controller design, selecting a suitable objective function is imperative for enhancing and optimizing parameter tuning. This objective function typically comprises sub-objective functions representing error indices related to frequency deviation, area control errors, and tie-line power deviation^21–23^. Prioritizing sub-objective functions and assigning appropriate ranks and weights are crucial for obtaining an optimal solution to the objective function. Subsequently, further optimization of the objective function is necessary once the weights have been determined.

This study employs the ROC technique as a systematic approach to evaluate the weights associated with sub-objective functions of tuning of proportional-integral-derivative (PID) controller for AGC problem of TAIPS. Controller tuning is achieved through Jaya optimization algorithm (JOA). JOA relies solely on the population size and the total number of iterations, devoid of any specific controlling parameters unique to the algorithm. Consequently, optimization processes become more accurate and less complex when employing the JOA^15^. Integral time absolute errors (ITAE) of frequency deviations and control errors for areas 1 and 2, along with ITAE of fluctuation in tie-line power, serve as sub-objective functions. The weighted sub-objective functions are then aggregated to form the comprehensive objective function. Differing from prior studies, this research adopts the ROC method to systematically determine the weights of sub-objective functions, departing from assigning equal or random values. To validate the efficacy of the JOA utilized in the proposed method, comparative analyses are presented through tables and response evaluations. The optimization results obtained from JOA are analysed against those from other optimization techniques, including teacher-learner based optimization algorithm (TLBOA), Luus-Jaakola algorithm (LJA), Nelder-Mead simplex algorithm (NMSA), elephant herding optimization algorithm (EHOA), and differential evolution algorithm (DEA). Furthermore, to assess applicability, six distinct case analyses under varying load deviations are considered. The primary highlights of this contribution include:

- ROC method is implemented for AGC problem of TAIPS. The ROC method facilitates a systematic assessment of weights associated with sub-objective functions.
- Based on the constructed objective function, which incorporates ITAEs of frequency deviations, control errors for areas 1 and 2, and fluctuation in tie-line power as sub-objective functions, a PID controller is designed.
- To minimize the objective function, JOA is employed in this study. The outcomes obtained from JOA are then compared with TLBOA, LJA, NMSA, EHOA, and DEA, for validation purposes.
- Furthermore, six case studies are conducted considering various load variations to evaluate the system’s responses to frequency fluctuations and tie-line exchange.
- Additionally, non-parametric and statistical analyses are employed to demonstrate the practical significance of JOA-based PID controller.

The paper is organized as follows: “ROC method” provides a brief introduction to the ROC method. In “System under consideration”, the model description of TAIPS is presented. “ROC based controller design” discusses the implementation of the ROC method for TAIPS, formulation of the objective function, and introduction of the JOA. In “Results and discussions”, the simulation outcomes are tabulated, discussed, and presented in the form of plots. Finally, “Conclusion” summarizes the conclusions drawn from the results and discussions.

## ROC method

ROC method offers a straightforward approach to determining weights^13^. It involves ranking multiple solutions (objectives) based on their significance and assigning weights accordingly. The essence of the ROC method lies in minimizing the errors associated with each weight by identifying the centroid of potential weights while preserving the ranking order of objectives. This method ensures a uniform distribution of weights across all objectives.

Consider a scenario where there are *N* sub-objectives contributing to an overall objective function. Let $\omega _M$ represent the weight of the $M{\text {th}}$ sub-objective, where $M=1,2,\ldots ,N$, and let $R_M$ denote its rank. The formula for weight determination using the ROC method is presented in ((1)).

1 $$\begin{aligned} \omega _M=\frac{1}{N}\sum _{M=1}^{N}\frac{1}{R_M}. \end{aligned}$$

The methodical procedure for employing the ROC method for weight determination is outlined below:

1. Step 1: Begin by identifying the sub-objective functions and prioritize them based on their significance.
2. Step 2: Associate weights to each of the sub-objective functions.
3. Step 3: Provide ranks to the sub-objective functions according to the priorities established in Step 1.
4. Step 4: Define the total number of sub-objectives as *N*, with the rank of the $M^{\text {th}}$ sub-objective denoted as $R_M$, and the weight of the $M^{\text {th}}$ sub-objective as $\omega _M$.
5. Step 5: Utilize the formula (1) to compute the weight of the sub-objective having $M=1$ by substituting the values of *N*, $R_M$, and $\omega _M$.
6. Step 6: After computation, increment the value of *M* by 1.
7. Step 7: Repeat Step 5 and Step 6 until the value of *M* equals *N*.

**Figure 1:**
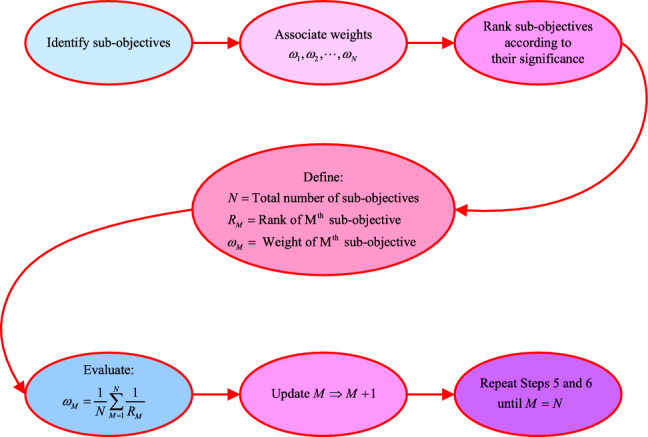
Stepwise implementation of ROC method.

The sequential execution of the ROC method for weight determination is depicted in Fig. 1.

## System under consideration

**Figure 2:**
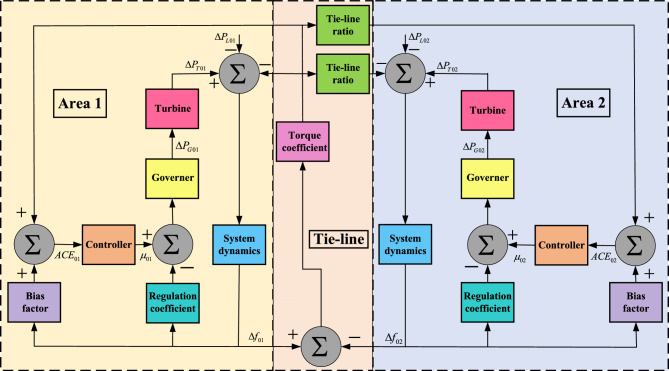
Two area interconnected power system.

The schematic representation of the TAIPS is illustrated in Fig. 2. This system configuration, outlined in^15^, encompasses two thermal power plants, each contributing 1000 MW to the total load, thereby establishing a combined capacity of 2000 MW. This setup mirrors a realistic interconnected power system. The transfer functions corresponding to the various blocks depicted in Fig. 2 are detailed below:

- Turbine’s transfer functions for area 1 and area 2 are given in (()) and ((3)), respectively.
  2 $$\begin{aligned} TF_{T01}= & {} \frac{1}{1+s\tau _{T01}} \end{aligned}$$
  3 $$\begin{aligned} TF_{T02}= & {} \frac{1}{1+s\tau _{T02}} \end{aligned}$$
- Generator’s transfer functions for area 1 and area 2 are given in ((4)) and ((5)), respectively.
  4 $$\begin{aligned} TF_{G01}= & {} \frac{1}{1+s\tau _{G01}} \end{aligned}$$
  5 $$\begin{aligned} TF_{G02}= & {} \frac{1}{1+s\tau _{G02}} \end{aligned}$$
- Transfer function of system dynamics for area 1 and area 2 are given in ((6)) and ((7)), respectively.
  6 $$\begin{aligned} TF_{01}=\frac{K_{01}}{1+s\tau _{01}} \end{aligned}$$
  7 $$\begin{aligned} TF_{02}= & {} \frac{K_{02}}{1+s\tau _{02}} \end{aligned}$$
- Transfer function for torque coefficient between area 1 and area 2 is given in ((8)).
  8 $$\begin{aligned} TF_{TC}=\frac{T_{0102}}{s} \end{aligned}$$

**Figure 3:**
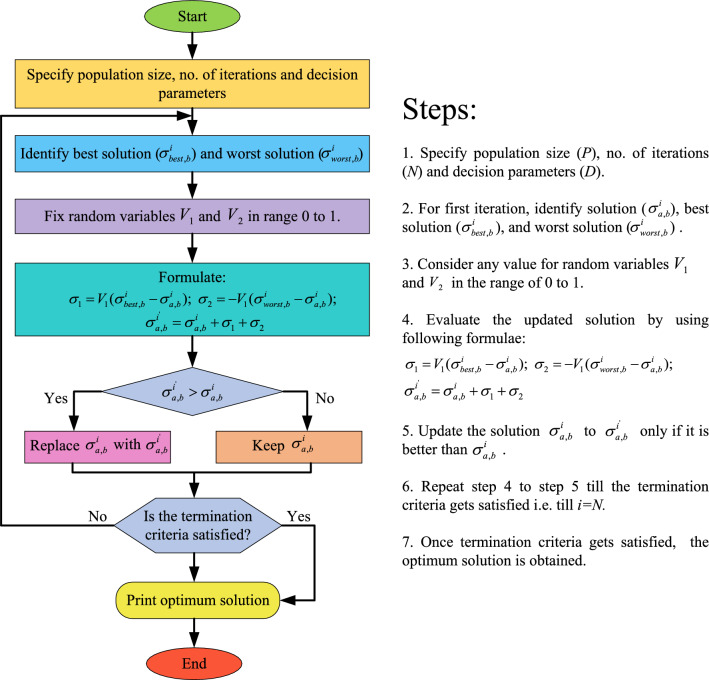
Steps and flowchart for JOA.

**Figure 4:**
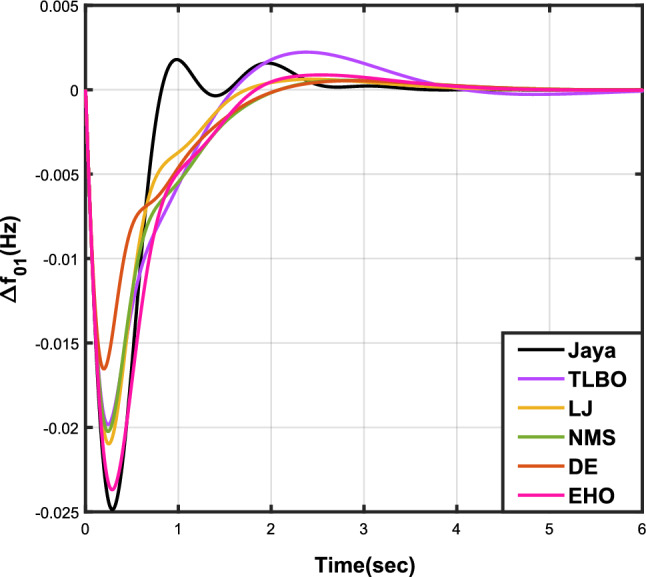
Case 1: Frequency fluctuations for area-1.

**Figure 5:**
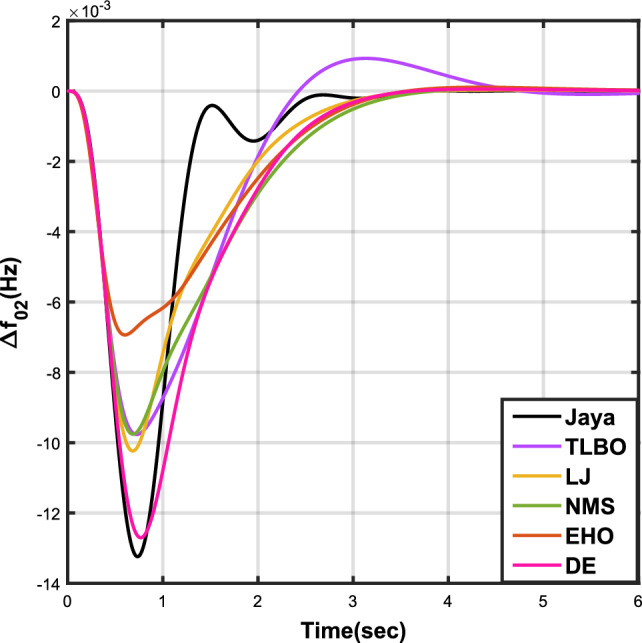
Case 1: Frequency fluctuations for area-2.

**Figure 6:**
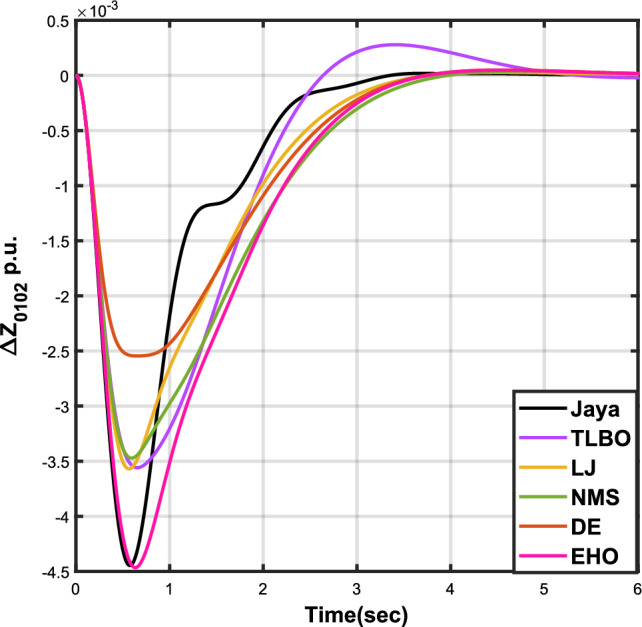
Case 1: Tie-line power fluctuation.

**Figure 7:**
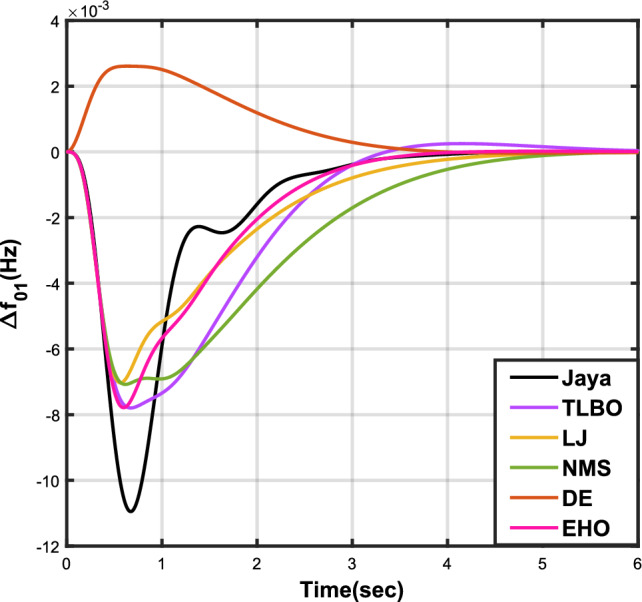
Case 2: Frequency fluctuations for area-1.

**Figure 8:**
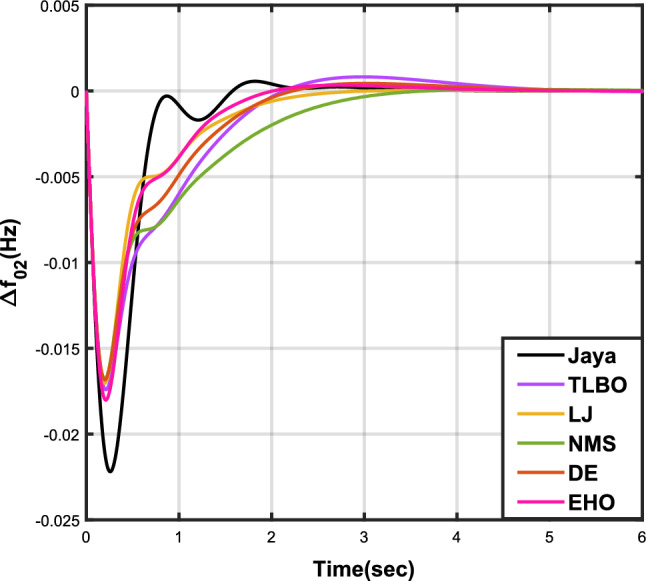
Case 2: Frequency fluctuations for area-2.

The variables used in Fig. 2 and (2)–(8) are tabulated in Table 1.

**Table 1:** TAIPS parameters and constraints.

TAIPS parameters					
Area 1			Area 2		
Parameter	Variable	Value (unit)	Parameter	Variable	Value (unit)
Frequency	*f*	60 Hz	Frequency	*f*	60 Hz
Frequency deviation	$\Delta f_{01}$	–	Frequency deviation	$\Delta f_{02}$	–
Area control error	$\text {ACE}_{01}$	–	Area control error	$\text {ACE}_{02}$	–
Bias factor	$\beta _{01}$	0.05 pu MW/Hz	Bias factor	$\beta _{02}$	0.05 pu MW/Hz
Control input	$\mu _{01}$	–	Control input	$\mu _{02}$	–
Governer’s speed regulation constant	$R_{01}$	2.4 Hz/pu	Governer’s speed regulation constant	$R_{02}$	2.4 Hz/pu
Governer’s time constant	$\tau _{G01}$	0.08 s	Governer’s time constant	$\tau _{G02}$	0.08 s
Turbine’s time constant	$\tau _{T01}$	0.3 s	Turbine’s time constant	$\tau _{T02}$	0.3 s
System’s gain constant	$K_{01}$	120 Hz/pu MW	System’s gain constant	$K_{02}$	120 Hz/pu MW
System’s time constant	$\tau _{01}$	20 s	System’s time constant	$\tau _{02}$	20 s
Governer power deviation	$\Delta P_{G01}$	–	Governer power deviation	$\Delta P_{G02}$	–
Turbine power deviation	$\Delta P_{T01}$	–	Turbine power deviation	$\Delta P_{T02}$	–
System’s load change	$\Delta P_{L01}$	–	System’s load change	$\Delta P_{L02}$	–

Tie-line					
Parameter	Variable	Value (unit)			
Torque coefficient	$T_{0102}$	0.545 pu			
Tie-line ratio	$A_{0102}$	− 1			
Tie-line power deviation	$\Delta Z_{0102}$	–			

Constraints					
Parameter	Max value	Min value			
Proportional gain	$\psi _P^{\text {max}}=3$	$\psi _P^{\text {min}}=0$			
Integral gain	$\psi _I^{\text {max}}=3$	$\psi _I^{\text {min}}=0$			
Derivative gain	$\psi _D^{\text {max}}=3$	$\psi _D^{\text {min}}=0$			
Filter	$F^{\text {max}}=500$	$F^{\text {min}}=0$			

## ROC based controller design

### PID controller

PID controllers have been widely employed in industrial and process control applications for many years. Renowned for their ease of tuning, straightforward structure, and user-friendly implementation, PID controllers remain a popular choice. A PID controller takes an error signal (*E*(*s*)) as input and produces a desired output (*U*(*s*)). Its general representation is provided in Eq. (9).

9 $$\begin{aligned} U(s)= \Bigg [\psi _P+\frac{\psi _{I}}{s}+\psi _{D}s\Bigg ]E(s) \end{aligned}$$

The PID controller utilized in this study integrates a filter *F* with the derivative gain to mitigate the impact of noise. Area control errors serve as inputs to the controller, while the generated outputs are control inputs. The controller representations for area 1 and area 2 are depicted in Eqs. (10) and (11), respectively.

10 $$\begin{aligned} \mu _{01}= & {} \Bigg [\psi _P+\frac{\psi _{I}}{s}+\psi _{D}\frac{1}{\frac{1}{s}+\frac{1}{N}}\Bigg ]ACE_{01} \end{aligned}$$

11 $$\begin{aligned} \mu _{02}= & {} \Bigg [\psi _P+\frac{\psi _{I}}{s}+\psi _{D}\frac{1}{\frac{1}{s}+\frac{1}{N}}\Bigg ]ACE_{02} \end{aligned}$$

The equations for $ACE_{01}$ and $ACE_{02}$ are presented in (12) and (13).

12 $$\begin{aligned} ACE_{01}(s)= & {} \Delta {Z_{0102}}(s)+\beta _{01}.\Delta {f_{01}}(s) \end{aligned}$$

13 $$\begin{aligned} ACE_{02}= & {} -A_{0102}.\Delta {Z_{0102}}(s)+\beta _{02}.\Delta {f_{02}}(s) \end{aligned}$$

### Objective function formulation

In ensuring a balanced power flow between area 1 and area 2, it is imperative to uphold their frequencies, minimize area control errors, and maintain tie-line powers. Considering these three aspects, an objective function is formulated. This objective function amalgamates three weighted sub-objective functions, each representing the integral time absolute error (ITAE) of the aforementioned concerns. Denoting these sub-objective functions as $X_1$, $X_2$, and $X_3$, their formulations are presented in Equations (14), (15), and (16).

14 $$\begin{aligned} X_{1}= & {} \int _0^{T_{st}}\Delta {f_{01}}tdt+\int _0^{T_{st}}\Delta {f_{02}}tdt \end{aligned}$$

15 $$\begin{aligned} X_{2}= & {} \int _0^{T_{st}}ACE_{01}tdt+\int _0^{T_{st}}ACE_{02}tdt \end{aligned}$$

16 $$\begin{aligned} X_{3}= & {} \int _0^{T_{st}}\Delta {Z_{0102}}tdt \end{aligned}$$

$X_1$ encompasses the ITAEs of frequency variation in area 1 and area 2, while $X_2$ and $X_3$ account for the ITAEs of area control errors in area 1 and area 2 and the ITAE of variation in tie-line power, respectively. The resultant objective function, formed by combining the weighted sub-objectives described in Eqs. (14), (15), and (16), is presented in Eq. (17).

17 $$\begin{aligned} X=\omega _1(X_{1})+\omega _2(X_{2})+\omega _3(X_{3}). \end{aligned}$$

Substituting values of $X_1$, $X_2$ and $X_3$ from (14), (15) and (16), respectively in (17), (17) is modified to (18).

18 $$\begin{aligned} X=\omega _1\Big (\int _0^{T_{st}}\Delta {f_{01}}tdt+\int _0^{T_{st}}\Delta {f_{02}}tdt\Big ) +\omega _2\Big (\int _0^{T_{st}}ACE_{01}tdt+\int _0^{T_{st}}ACE_{02}tdt) + \omega _3\Big (\int _0^{T_{st}}\Delta {Z_{0102}}tdt\Big ) \end{aligned}$$

The weights $\omega _1$, $\omega _2$, and $\omega _3$ are determined using Eq. (1), as explained in “Introduction”. Since there are three sub-objectives, *N* in Eq. (1) is 3, and *M* takes values from 1 to 3. The values of $\omega _1$, $\omega _2$, and $\omega _3$ are calculated as follows:

19 $$\begin{aligned} \begin{array}{cc} &{} M=1, \omega _1=\frac{1}{3}\Bigg [1+\frac{1}{2}+\frac{1}{3}\Bigg ] =0.61\\ &{} M=2, \omega _2=\frac{1}{3}\Bigg [\frac{1}{2}+\frac{1}{3}\Bigg ]=0.28\\ &{} M=3, \omega _3=\frac{1}{3}\Bigg [\frac{1}{3}\Bigg ]=0.11\\ \end{array} \end{aligned}$$

Substituting values of $\omega _1$, $\omega _2$ and $\omega _3$ from (19) in (18), overall objective function is modified to (20).

20 $$\begin{aligned} X=0.61\Big (\int _0^{T_{st}}\Delta {f_{01}}tdt+\int _0^{T_{st}}\Delta {f_{02}}tdt\Big )+ 0.28\Big (\int _0^{T_{st}}ACE_{01}tdt+\int _0^{T_{st}}ACE_{02}tdt) + 0.11\Big (\int _0^{T_{st}}\Delta {Z_{0102}}tdt\Big ) \end{aligned}$$

To minimize the objective function, this article employs the JOA, as discussed in “Jaya optimization algorithmJayaalgorithm”. Constraints are imposed on the controller parameters to confine the search space during minimization. These constraints are expressed in (21), and their specific values are presented in Table 1.

21 $$\begin{aligned} \begin{array}{cc} &{} \psi _P^{min}<\psi _P<\psi _P^{max}\\ &{} \psi _I^{min}<\psi _I<\psi _I^{max}\\ &{} \psi _D^{min}<\psi _D<\psi _D^{max}\\ &{} F^{min}<F<F^{max} \end{array} \end{aligned}$$

### Jaya optimization algorithm

The Jaya optimization algorithm (JOA), initially devised to handle both constrained and unconstrained optimization problems, draws its inspiration from the concept of “victory”, symbolized as “Jaya” in Sanskrit^24,25^. Mimicking the principle of “survival of the fittest” observed in nature, solutions within the Jaya population tend to converge towards the global optimum by discarding less suitable solutions. Remarkably, this algorithm operates solely based on the total number of iterations and the population size, obviating the need for specific controlling parameters.

Let *N* represents the termination criteria, *P* denote the population size, and *D* indicate the total number of decision parameters. In JOA, a solution is denoted as $\sigma _{a,b}$, where $a=1,2,\ldots , P$ represents the population and $b=1,2,\ldots , D$ signifies the decision parameters. The updated solution for the $a^{\text {th}}$ population and the $b^{\text {th}}$ decision parameter at the $i^{\text {th}}$ iteration is given by

22 $$\begin{aligned} \sigma ^{i^{'}}_{a,b}=\sigma ^{i}_{a,b}+\sigma _{1}+\sigma _{2} \end{aligned}$$

where,

23 $$\begin{aligned} \left. \begin{array}{ll} \sigma _{1}=V_{1}(\sigma ^{i}_{best,b}-\sigma ^{i}_{a,b})\\ \sigma _{2}=-V_{2}(\sigma ^{i}_{worst,b}-\sigma ^{i}_{a,b}) \\ \end{array} \right\} \end{aligned}$$

In (23), the random variables $V_1$ and $V_2$ are introduced, each ranging from 0 to 1, to facilitate the algorithm’s exploration. Following each iteration, an updated solution is generated. This updated solution is deemed acceptable for further iterations only if it outperforms the previous solution. A visual representation of the steps involved in the JOA, along with the procedural details, is provided in Fig. 3.

## Results and discussions

In this section, we conduct an analysis of AGC problem within the framework of TAIPS. The objective is to address three distinct goals by integrating them into a unified objective function (20), with their respective weights determined using ROC technique. Subsequently, the minimization of (20) is undertaken utilizing JOA, while adhering to the constraints outlined in (21). To evaluate the effectiveness and applicability of the proposed methodology, six different case studies, as detailed in Table 2, are simulated in the time domain, encompassing various load conditions. The simulation results encompass fitness values of the objective and sub-objective functions (*X*, $X_1$, $X_2$, and $X_3$), decision parameters pertaining to controller settings ($\psi _P$, $\psi _I$, $\psi _D$, and *F*), settling times for $\Delta {f_{01}}$, $\Delta {f_{02}}$, and $\Delta {Z_{0102}}$, as well as their peak overshoots. In order to validate the simulation outcomes of the JOA-based PID controller, we include PID controllers optimized using alternative algorithms such as TLBOA, LJA, NMSA, DEA, and EHOA. Furthermore, to substantiate the validation, a comprehensive statistical analysis is conducted for the JOA, TLBOA, LJA, NMSA, DEA, and EHOA-based PID controllers. This analysis compares their mean, minimum, and maximum values, along with their respective standard deviations. Finally, a Friedman rank test is performed across all the algorithms, computing their mean ranks, *Q* value, and *p* value for comparison and validation purposes.

**Table 2:** Case analysis.

Case analysis	Step load variations	
	Area 1	Area 2
I	0.025	0
II	0	0.025
III	0.025	0.025
IV	0.025	− 0.025
V	0.025	0.05
VI	0.05	0.025

The outcomes of case analysis I are systematically arranged and displayed in Table 3. Visual representations of frequency deviations for area-1 ($\Delta {f_{01}}$) and area-2 ($\Delta {f_{02}}$), as well as tie-line power deviation ($\Delta {Z_{0102}}$), are depicted in Figs. 4, 5, and 6, respectively. These visualizations clearly indicate the superior performance of the JOA-based PID controller over other PID controllers. The enhanced response of the JOA-based PID controller is evident from the graphical representations. This observation is corroborated by the quantitative analysis presented in Table 3, which highlights that the PID controller employing the JOA achieves the fastest settling time compared to PID controllers utilizing alternative algorithms. Notably, the JOA-based PID controller achieves the optimal outcome, as evidenced by the minimum value of the objective function *X*, underscoring its efficacy in enhancing system performance. Similarly, the utilization of the JOA-based PID controller results in the minimum values of the sub-objective functions $X_{1}$, $X_{2}$, and $X_{3}$, further emphasizing its effectiveness.

**Table 3:** Results for case analysis I.

		Jaya	TLBO	LJ	NMS	DE	EHO
Fitness	*X*	0.02871	0.05415	0.03766	0.06752	0.04271	0.04320
	$X_{1}$	0.04366	0.08484	0.05700	0.10319	0.06622	0.06706
	$X_{2}$	0.01257	0.02060	0.01667	0.02834	0.01748	0.01755
	$X_{3}$	0.01877	0.03415	0.02491	0.04451	0.02692	0.02722
Decision parameters	$\psi _P$	2.16059	1.76926	2.87764	2.13088	2.15090	2.22966
	$\psi _I$	2.99912	2.49640	2.87080	2.00520	2.67464	2.72155
	$\psi _D$	0.64783	1.15718	1.14092	1.24553	1.12530	1.20696
	F	470.811	433.201	273.225	177.700	347.197	349.361
Settling time (s)	$\Delta {f_{01}}$	1.96728	4.16135	2.25437	3.00036	3.51075	3.63759
	$\Delta {f_{02}}$	3.29440	5.00388	4.36248	4.88952	3.46296	3.48559
	$\Delta {{Z}_{0102}}$	3.44821	5.41281	4.49584	4.99229	3.64359	3.65955
Peak overshoots (p.u.)	$\Delta {f_{01}}$	0.06215	0.04876	0.04771	0.04713	0.04895	0.04716
	$\Delta {f_{02}}$	0.03065	0.02182	0.01968	0.01982	0.02127	0.01997
	$\Delta {{Z}_{0102}}$	0.01031	0.00824	0.00687	0.00779	0.00768	0.00730

**Figure 9:**
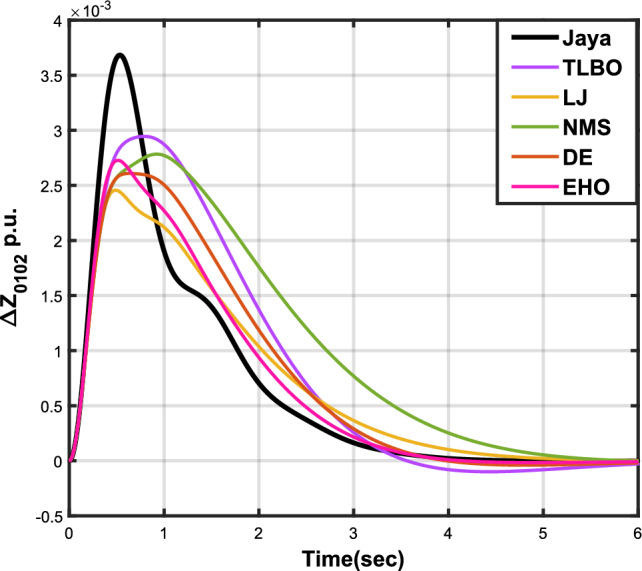
Case 2: Tie-line power fluctuation.

Table 4 presents the simulation results obtained from case analysis II. The graphical responses of $\Delta {f_{01}}$, $\Delta {f_{02}}$, and $\Delta {Z_{0102}}$ are depicted in Figs. 7, 8, and 9, respectively. As observed in case analysis I, the graphical representations in this analysis also indicate the superiority of the JOA-based PID controller over other PID controllers. Quantitative analysis reveals that the PID controller utilizing the JOA achieves the quickest settling time compared to controllers employing alternative algorithms. Furthermore, the JOA-based PID controller attains the optimal outcome, as evidenced by the lowest value of the overall objective function.

**Table 4:** Results for case analysis II.

		Jaya	TLBO	LJ	NMS	DE	EHO
Fitness	*X*	0.01184	0.02270	0.01556	0.02803	0.01802	0.01412
	$X_{1}$	0.01559	0.03030	0.02035	0.03683	0.02395	0.01874
	$X_{2}$	0.00449	0.00735	0.00595	0.01012	0.00627	0.00514
	$X_{3}$	0.00670	0.01219	0.00889	0.015897	0.00972	0.00759
Decision parameters	$\psi _P$	2.16059	1.76926	2.87764	2.13088	2.22966	2.41632
	$\psi _I$	2.99912	2.49640	2.87080	2.00520	2.72155	2.98598
	$\psi _D$	0.64783	1.15718	1.14092	1.24553	1.20696	1.05261
	F	470.811	433.201	273.225	177.700	349.360	198.015
Settling time (s)	$\Delta {f_{01}}$	3.29437	5.00398	4.36258	4.88862	3.48559	3.45161
	$\Delta {f_{02}}$	1.96760	4.16162	2.25420	2.99960	3.63764	1.79290
	$\Delta {Z_{0102}}$	3.44822	5.41279	4.49594	4.99236	3.65956	3.61091
Peak overshoots (p.u.)	$\Delta {f_{01}}$	0.01095	0.00779	0.00703	0.00707	0.00713	0.00778
	$\Delta {f_{02}}$	0.02219	0.01741	0.01704	0.01681	0.01684	0.01802
	$\Delta {Z_{0102}}$	0.00368	0.00294	0.00245	0.00278	0.00260	0.00272

**Figure 10:**
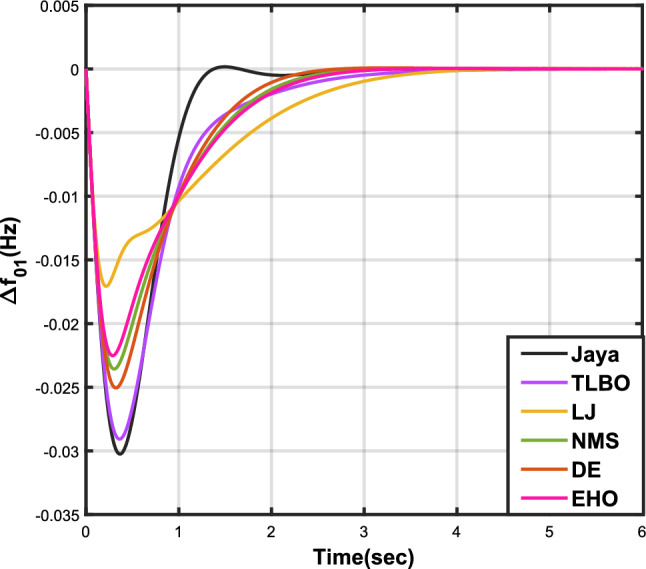
Case 3: Frequency fluctuations for area-1.

**Figure 11:**
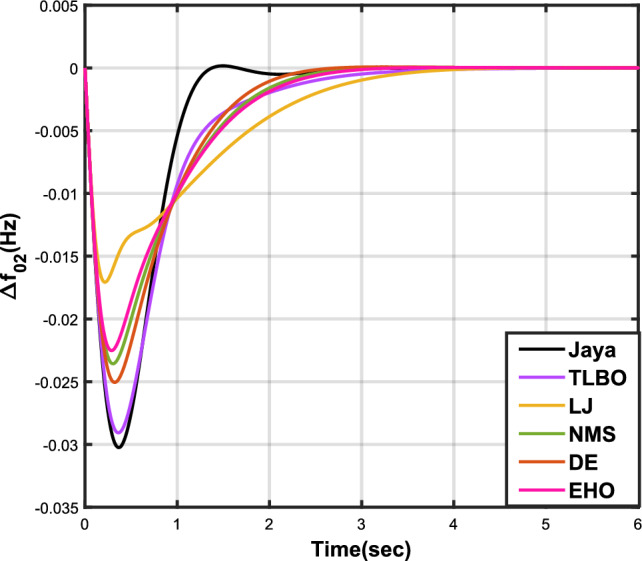
Case 3: Frequency fluctuations for area-2.

**Figure 12:**
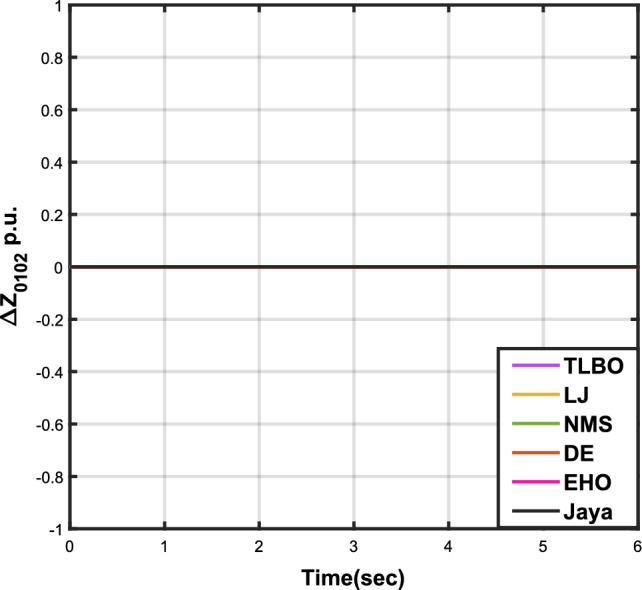
Case 3: Tie-line power fluctuation.

In Table 5, the results of case analysis III are presented. It is evident from the findings that the JOA-based PID controller outperformed all other controllers based on different algorithms. The JOA-based PID controller exhibited the lowest values for both the objective function and sub-objective functions, and it also demonstrated the fastest settling time. This superior performance of the JOA-based PID controller is illustrated through graphical representations in Figs. 10, 11, and 12, which depict $\Delta {f_{01}}$, $\Delta {f_{02}}$, and $\Delta {Z_{0102}}$ respectively.

**Table 5:** Results for case analysis III.

		Jaya	TLBO	LJ	NMS	DE	EHO
	*X*	0.01561	0.02777	0.03377	0.02350	0.02201	0.02412
	$X_{1}$	0.02141	0.03810	0.04632	0.03224	0.03019	0.03309
	$X_{2}$	0	0	0	0	0	0
	$X_{3}$	0.00910	0.01619	0.01968	0.01370	0.01283	0.01406
Fitness Decision parameters	$\psi _P$	1.59717	1.63668	2.67985	1.93034	1.77656	2.06715
	$\psi _I$	2.88401	2.32600	2.63987	2.68327	2.67970	2.72281
	$\psi _D$	0.43334	0.48760	1.32552	0.76246	0.68293	0.82655
	F	218.226	369.819	440.721	300.938	321.602	228.822
Settling time (s)	$\Delta {f_{01}}$	1.26864	2.87640	3.58554	2.47070	2.23259	2.61891
	$\Delta {f_{02}}$	1.26864	2.87640	3.58554	2.47070	2.23259	2.61891
	$\Delta {Z_{0102}}$	0	0	0	0	0	0
Peak overshoots (p.u.)	$\Delta {f_{01}}$	0.03024	0.02907	0.01707	0.02356	0.02505	0.02252
	$\Delta {f_{02}}$	0.03024	0.02907	0.01707	0.02356	0.02505	0.02252
	$\Delta {Z_{0102}}$	0	0	0	0	0	0

**Figure 13:**
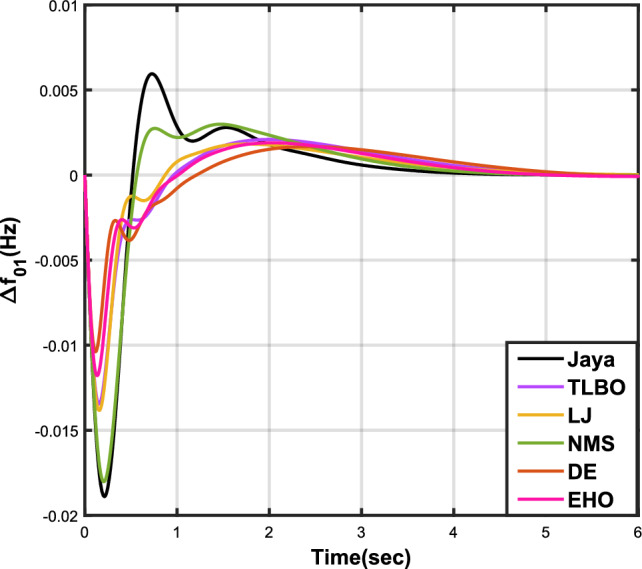
Case 4: Frequency fluctuations for area-1.

**Figure 14:**
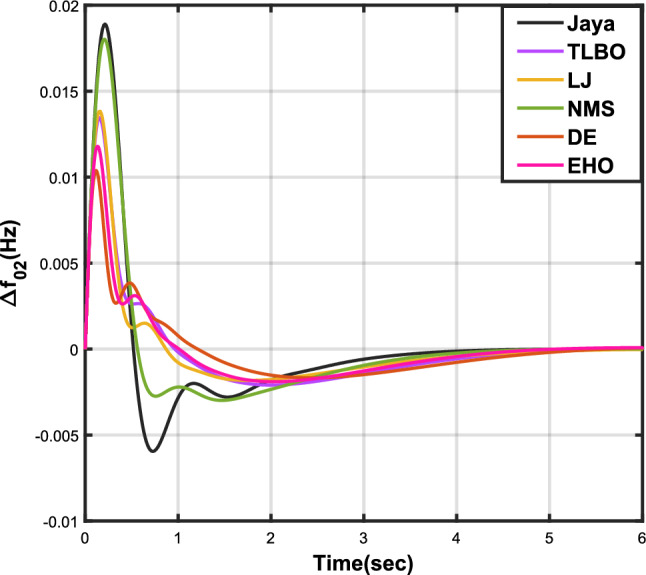
Case 4: Frequency fluctuations for area-2.

**Figure 15:**
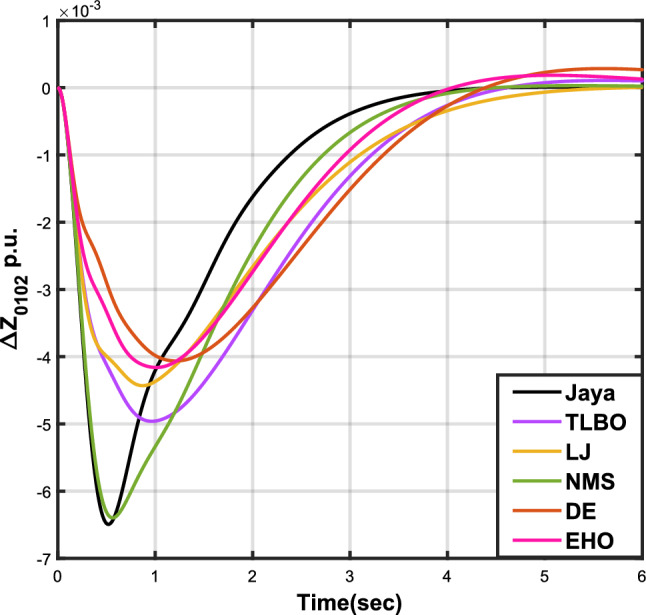
Case 4: Tie-line power fluctuation.

The results for case analysis IV are presented in Table 6, accompanied by graphical representations of $\Delta {f_{01}}$, $\Delta {f_{02}}$, and $\Delta {Z_{0102}}$ in Figs. 13, 14, and 15 respectively, providing a visual understanding of the data. As observed in Table 6, the JOA-based PID controller exhibits the shortest settling time among PID controllers using different algorithms. Additionally, it achieves the lowest values for the sub-objective functions *X*, $X_{1}$, and $X_{2}$, as well as for the objective function $X_{3}$.

**Table 6:** Results for case analysis IV.

		Jaya	TLBO	LJ	NMS	DE	EHO
Fitness	*X*	0.02141	0.03810	0.04632	0.03224	0.03019	0.03309
	$X_{1}$	0.02128	0.02759	0.02196	0.02516	0.029657	0.02430
	$X_{2}$	0.00958	0.01811	0.01506	0.01322	0.02038	0.01496
	$X_{3}$	0.01272	0.02820	0.02271	0.01866	0.03393	0.02372
Decision parameters	$\psi _P$	2.29451	2.33687	2.94046	1.97933	2.74871	2.79796
	$\psi _I$	2.97746	2.35083	2.60156	2.44598	2.74842	2.98638
	$\psi _D$	0.80115	1.67075	1.57187	0.91227	2.74868	2.14128
	F	251.695	428.201	269.611	433.240	375.556	410.815
Settling time (s)	$\Delta {f_{01}}$	3.32060	4.55333	4.39178	3.73260	4.96781	4.34881
	$\Delta {f_{02}}$	3.32060	4.55333	4.39178	3.73260	4.96781	4.34881
	$\Delta {Z_{0102}}$	3.58304	6.12057	4.86206	3.85087	7.81936	6.56500
Peak overshoots (p.u.)	$\Delta {f_{01}}$	0.01890	0.01346	0.01383	0.01803	0.01039	0.01179
	$\Delta {f_{02}}$	0.01890	0.01346	0.01383	0.01803	0.01039	0.01179
	$\Delta {Z_{0102}}$	0.00649	0.00496	0.00443	0.00640	0.00406	0.00416

**Figure 16:**
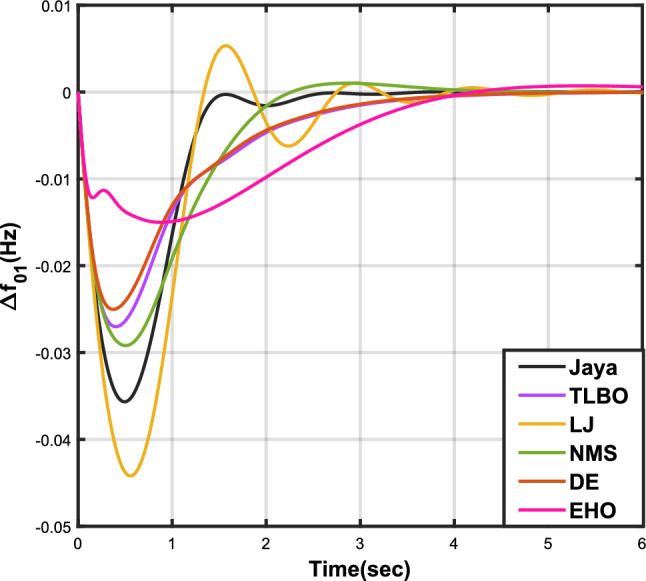
Case 5: Frequency fluctuations for area-1.

**Figure 17:**
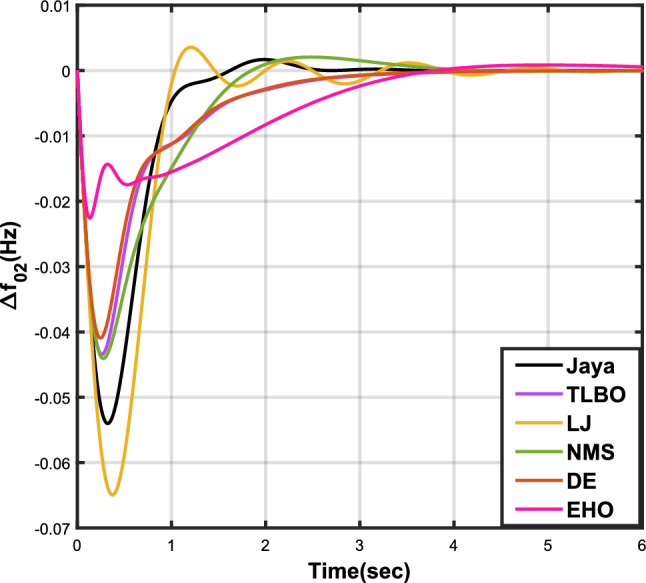
Case 5: Frequency fluctuations for area-2.

**Figure 18:**
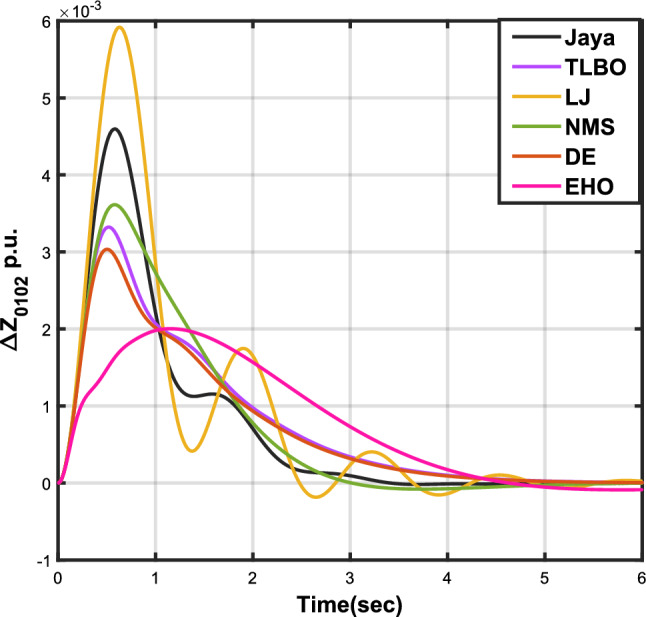
Case 5: Tie-line power fluctuation.

Table 7 provides an overview of the findings from case analysis V, while the corresponding responses for $\Delta {f_{01}}$, $\Delta {f_{02}}$, and $\Delta {Z_{0102}}$ are illustrated in Figs. 16, 17, and 18, respectively. The results highlight the superior performance of the JOA-based PID controller compared to controllers based on other algorithms. Specifically, the JOA-based PID controller achieved the lowest values for both the objective and sub-objective functions, while also demonstrating the shortest settling time. These outcomes underscore its effectiveness relative to the other controllers considered.

**Table 7:** Results for case analysis V.

		Jaya	TLBO	LJ	NMS	DE	EHO
Fitness	*X*	0.02719	0.04401	0.04942	0.04141	0.04113	0.08085
	$X_{1}$	0.03667	0.05946	0.06631	0.05616	0.05557	0.10959
	$X_{2}$	0.00420	0.00604	0.00651	0.00511	0.00565	0.00935
	$X_{3}$	0.01557	0.02527	0.02948	0.02354	0.02362	0.04632
Decision parameters	$\psi _P$	1.57790	2.41757	1.24749	1.61030	2.62420	2.94945
	$\psi _I$	2.86765	2.67030	2.31084	2.83715	2.84232	2.71181
	$\psi _D$	0.49063	0.74965	0.30254	0.80291	0.84047	2.58650
	F	456.862	453.404	267.357	406.203	423.436	416.925
Settling time (s)	$\Delta {f_{01}}$	2.35130	3.88179	3.71026	3.53821	3.84897	7.09332
	$\Delta {f_{02}}$	2.23253	2.93474	3.04536	3.38719	2.89593	6.41211
	$\Delta {Z_{0102}}$	3.01128	4.31432	4.04959	4.04802	4.29372	7.51010
Peak overshoots (p.u.)	$\Delta {f_{01}}$	0.03567	0.02702	0.04420	0.02919	0.02503	0.01500
	$\Delta {f_{02}}$	0.05401	0.04343	0.06495	0.04406	0.04094	0.02259
	$\Delta {Z_{0102}}$	0.00460	0.00332	0.00592	0.00361	0.00303	0.00200

**Figure 19:**
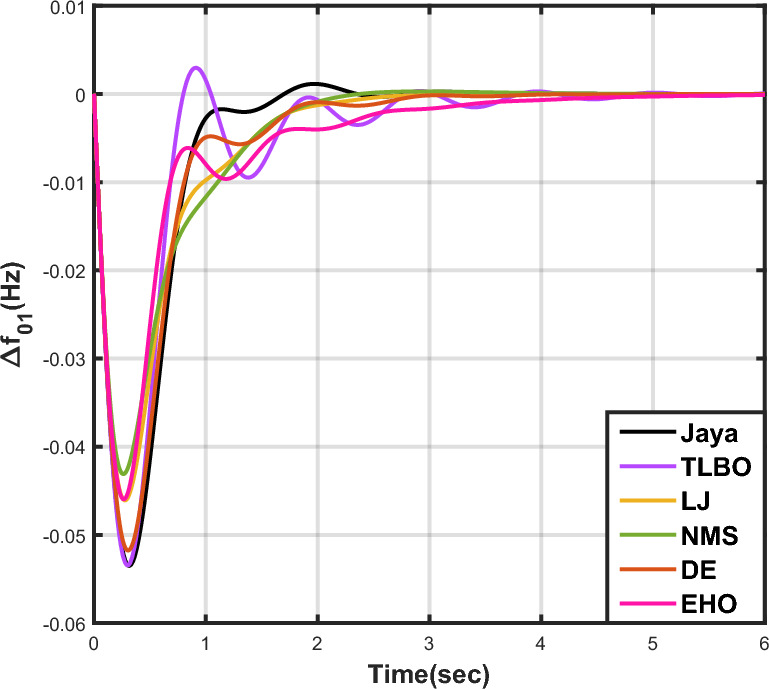
Case 6: Frequency fluctuations for area-1.

**Figure 20:**
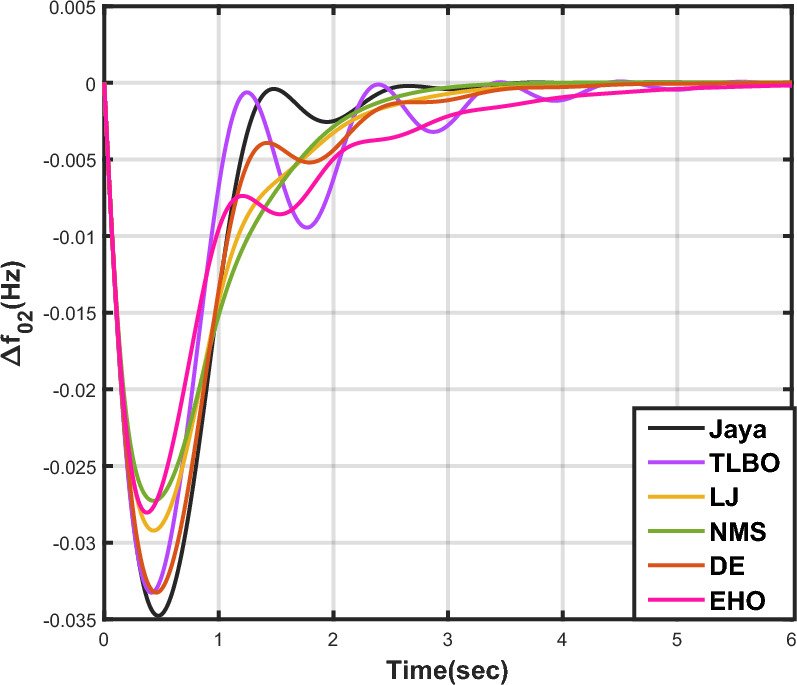
Case 6: Frequency fluctuations for area-2.

**Figure 21:**
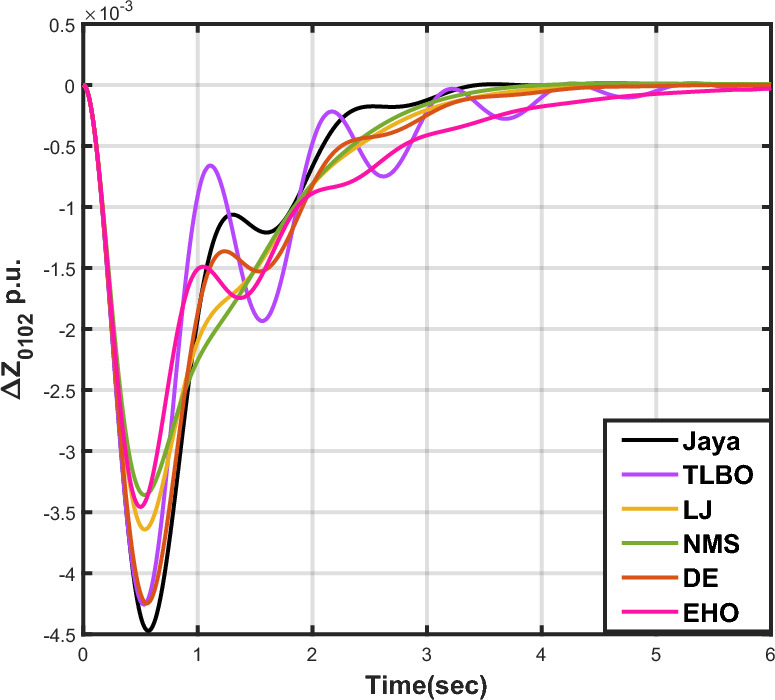
Case 6: Tie-line power fluctuation.

The results for case analysis VI are presented in Table 8, while the corresponding responses for $\Delta {f_{01}}$, $\Delta {f_{02}}$, and $\Delta {Z_{0102}}$ are depicted in Figs. 19, 20, and 21, respectively. As observed in previous cases, the JOA-based PID controller consistently achieves the lowest values for both the objective and sub-objective functions, as well as the shortest settling time across all three responses. This reaffirms its superiority over the other controllers.

**Table 8:** Results for case analysis VI.

		Jaya	TLBO	LJ	NMS	DE	EHO
Fitness	*X*	0.02579	0.04125	0.03346	0.03228	0.03504	0.05286
	$X_{1}$	0.03467	0.05545	0.04514	0.04373	0.04728	0.07147
	$X_{2}$	0.00403	0.00553	0.00499	0.00479	0.00517	0.00690
	$X_{3}$	0.01499	0.02434	0.01920	0.01812	0.02010	0.03037
Decision parameters	$\psi _P$	1.73345	2.45255	2.10758	2.10018	1.97645	2.85085
	$\psi _I$	2.97574	2.83102	2.82148	2.92659	2.69841	2.64804
	$\psi _D$	0.48790	0.41136	0.67913	0.79286	0.50773	0.61215
	F	241.425	192.983	307.822	403.370	423.315	308.163
Settling time (s)	$\Delta {f_{01}}$	2.00491	3.57692	2.19407	2.03995	2.56470	3.56396
	$\Delta {f_{02}}$	2.39376	4.16908	3.12192	2.77544	3.29122	4.63191
	$\Delta {Z_{0102}}$	3.09728	4.87530	3.59863	3.36761	3.70750	5.05689
Peak overshoots (p.u.)	$\Delta {f_{01}}$	0.05351	0.05344	0.04607	0.04310	0.05177	0.04595
	$\Delta {f_{02}}$	0.03477	0.03327	0.02922	0.02727	0.03326	0.02804
	$\Delta {Z_{0102}}$	0.00447	0.00426	0.00364	0.00336	0.00425	0.00346

A statistical comparison is conducted among algorithms based on JOA, TLBOA, LJA, NMSA, DEA, and EHOA across all six case studies. Table 9 presents the results of this analysis, including mean values ($X_{Mean}$), minimum values ($X_{Min}$), maximum values ($X_{Max}$), and standard deviations ($X_{SD}$). Upon comparing the results across all algorithms, it becomes evident that the JOA-based controller consistently outperforms TLBOA, LJA, NMSA, DEA, and EHOA algorithms. Across case studies I to VI, the JOA consistently yields the lowest mean and minimum values. Furthermore, the standard deviations associated with the JOA algorithm are the lowest among all six cases, indicating its superior performance and reliability.

**Table 9:** Statistical analysis.

Cases	Statistical measures	Jaya	TLBO	LJ	NMS	DE	EHO
I	$X_{Mean}$	0.01288	0.04039	0.02418	0.03091	0.02003	0.03148
	$X_{Min}$	0.01225	0.02573	0.01588	0.02034	0.01701	0.02202
	$X_{Max}$	0.01390	0.05881	0.02943	0.04373	0.02158	0.03784
	$X_{SD}$	0.00066	0.01227	0.00633	0.00948	0.00178	0.00729
II	$X_{Mean}$	0.01236	0.04208	0.02726	0.03531	0.02118	0.02863
	$X_{Min}$	0.01188	0.02271	0.01556	0.02804	0.01802	0.01413
	$X_{Max}$	0.01382	0.05721	0.04132	0.05300	0.02615	0.04722
	$X_{SD}$	0.00032	0.01291	0.01109	0.01074	0.00342	0.01270
III	$X_{Mean}$	0.02751	0.04245	0.06347	0.02994	0.04614	0.03676
	$X_{Min}$	0.01561	0.02778	0.03377	0.02350	0.02201	0.02412
	$X_{Max}$	0.03373	0.05305	0.09527	0.08924	0.03495	0.04847
	$X_{SD}$	0.00397	0.01057	0.02912	0.02789	0.00597	0.00879
IV	$X_{Mean}$	0.01927	0.03884	0.03110	0.03677	0.03073	0.03132
	$X_{Min}$	0.01759	0.02672	0.02141	0.02203	0.02983	0.02311
	$X_{Max}$	0.02079	0.05984	0.03661	0.04879	0.03318	0.03913
	$X_{SD}$	0.00136	0.01425	0.00609	0.01070	0.00138	0.00623
V	$X_{Mean}$	0.03545	0.08731	0.07835	0.09125	0.08055	0.09417
	$X_{Min}$	0.02719	0.04401	0.04942	0.04141	0.04113	0.08085
	$X_{Max}$	0.05501	0.12775	0.14871	0.14832	0.10149	0.12126
	$X_{SD}$	0.01139	0.03617	0.04159	0.03834	0.02454	0.01568
VI	$X_{Mean}$	0.03368	0.07293	0.06080	0.07426	0.05033	0.08873
	$X_{Min}$	0.02579	0.04125	0.03346	0.03228	0.03504	0.05286
	$X_{Max}$	0.03992	0.11652	0.08347	0.12428	0.06271	0.12256
	$X_{SD}$	0.00531	0.02814	0.01820	0.03302	0.01161	0.03083

A Friedman rank test is employed for non-parametric analysis to compare the optimization performances of the JOA, TLBOA, LJA, NMSA, DEA, and EHOA algorithms. This test assesses the mean rank of each algorithm, along with the overall *Q* and *p* values for all algorithms. The algorithm with a mean rank of 1 is considered to have the best performance. For the test to be validated, the *Q* value must be positive and the *p* value must be less than 5%.

Table 10 presents the mean ranks for each algorithm, as well as the overall *Q* value and *p* value. Among the algorithms JOA, TLBOA, LJA, NMSA, DEA, and EHOA, their respective mean ranks are 1, 3.83333, 4, 3.83333, 3.16667, and 5.16667. It is evident from these values that the JOA algorithm outperforms the others, having the lowest mean rank of 1. This finding is further supported by a positive *Q* value of 16.47619 and a *p* value of 0.005608, which is significantly lower than the 5%.

**Table 10:** Friedman rank test.

Friedman rank test						
	Jaya	TLBO	LJ	NMS	DE	EHO
Mean rank	1	3.83333	4	3.83333	3.166667	5.166667
*Q* value	*Q*=16.47619					
*p* value	*p*=0.005608					

## Conclusion

MCDM techniques are widely acknowledged for their effectiveness in various applications. This paper employed ROC method, a type of MCDM technique, to determine the weights of objective functions. These objective functions are derived from ITAE evaluations of frequency deviations, control errors, lie-line power deviation for AGC problem of a two-area power system. Using these objective functions, a PID controller is designed. The JOA algorithm is then employed to minimize the objective function. The system’s performance is evaluated under six different load variations.

To demonstrate the effectiveness of the JOA algorithm-based controller, optimization is also conducted using TLBOA, LJA, NMSA, DEA, and EHOA. Their outcomes are compared for each of the six load variations, with comparisons presented in both graphical and tabular formats. Key metrics considered for comparison include objective function values, decision parameters, settling time, and peak overshoots. The results indicated that the JOA algorithm consistently outperforms the other algorithms for all considered load variations. Additionally, statistical analysis and a Friedman rank test confirm the superiority of the JOA algorithm-based PID controller over other controllers. In future, fractional order controllers^26,27^, fuzzy controllers^28–33^ and learning based controllers^34,35^, along with learning based optimization techniques^36^, can be implemented for optimal generation control.

## Data Availability

The datasets used and/or analysed during the current study available from the corresponding author on reasonable request.
